# Application progress of human amniotic membrane in vitreoretinopathy: a literature review

**DOI:** 10.3389/fmed.2023.1206577

**Published:** 2023-10-10

**Authors:** Huawei Yang, Ziyue Li, Wei Jin, Anhuai Yang

**Affiliations:** ^1^Eye Center, Renmin Hospital of Wuhan University, Wuhan, China; ^2^First Clinical College, Wuhan University, Wuhan, China

**Keywords:** amniotic membrane, posterior segment of the eye, retinal hole, retinal detachment, retinitis pigmentosa, age-related macular degeneration, optic disc pit, choroidal hole

## Abstract

Recently, the application of the amniotic membrane (AM) in ophthalmology is gradually expanding from the anterior to the posterior segment of the eye. Its characteristics of anti-inflammation, anti-bacterial, anti-vascularization, immune regulation, anti-fibrosis, pro-epithelialization, and so forth have made it a hot topic in ophthalmic research. AM has been confirmed to repair photoreceptors, restore normal retinal structures, and close the abnormal structures in the optic disc. Currently, the application areas mainly include retinal hole, retinal detachment, optic disc pit, retinal degenerative diseases, and choroidal hole. This article reviews the current literature applying AM transplantation in the treatment of various posterior segment diseases while comparing the clinical outcomes with other techniques.

## 1. Introduction

**Figure 1:**
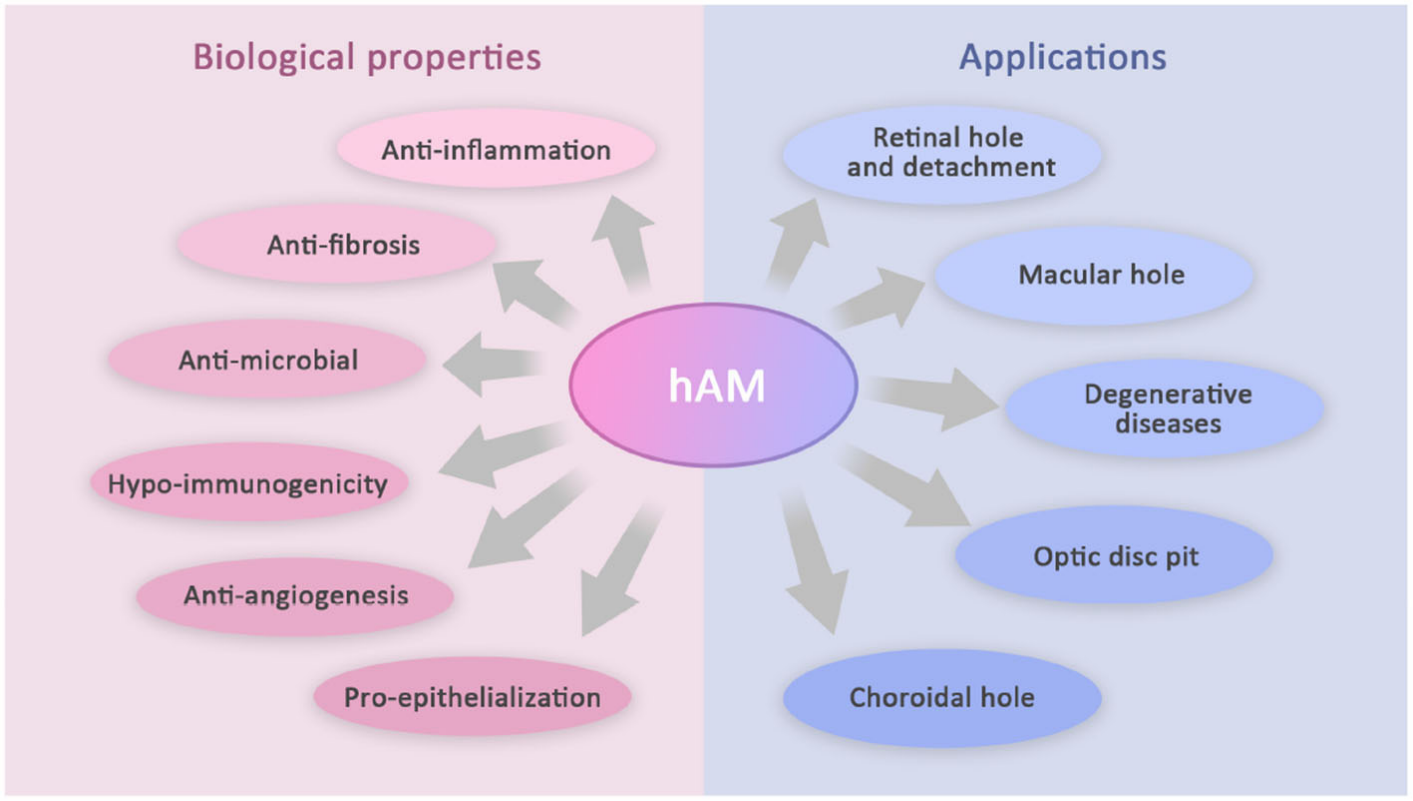
Biological properties and applications of hAM in vitreoretinopathy.

Human amniotic membrane (hAM) is the innermost layer of the placenta. It can be applied to various clinical settings considering its unique structure, biocompatibility, and other biological properties. Previous studies have shown its ability to reconstruct the corneal surface in cornea diseases, such as persistent epithelial defects, partial limbal stem-cell defects, bullous keratopathy, and corneal ulcers. hAM, as an effective conjunctival substitute, has also been used in the resection of pterygium, conjunctival diseases, and repair of conjunctival defects. Moreover, AM has been confirmed to repair photoreceptors, restore normal retinal structures, and close the abnormal structures in the optic disc. Its utility is being explored in some other challenging posterior segment diseases, of which this study will introduce the progress (Figure 1).

## 2. Materials and methods

In this literature review, a non-systematic search was carried out in PubMed, Web of Science, Embase, and Google scholar using the following query conditions: “[(eye) OR (ocular) OR (fundus)] AND [(amnion) OR (amniotic membrane)] NOT [(cornea) OR (surface)]”. Articles in English or with English abstracts were retrieved. Most relevant results concerning the posterior segment of the eye were manually screened and categorized by Yang HW. The retrieval scope included all applicable literature present in the above databases without temporal limits.

## 3. The structure of hAM

**Figure 2:**
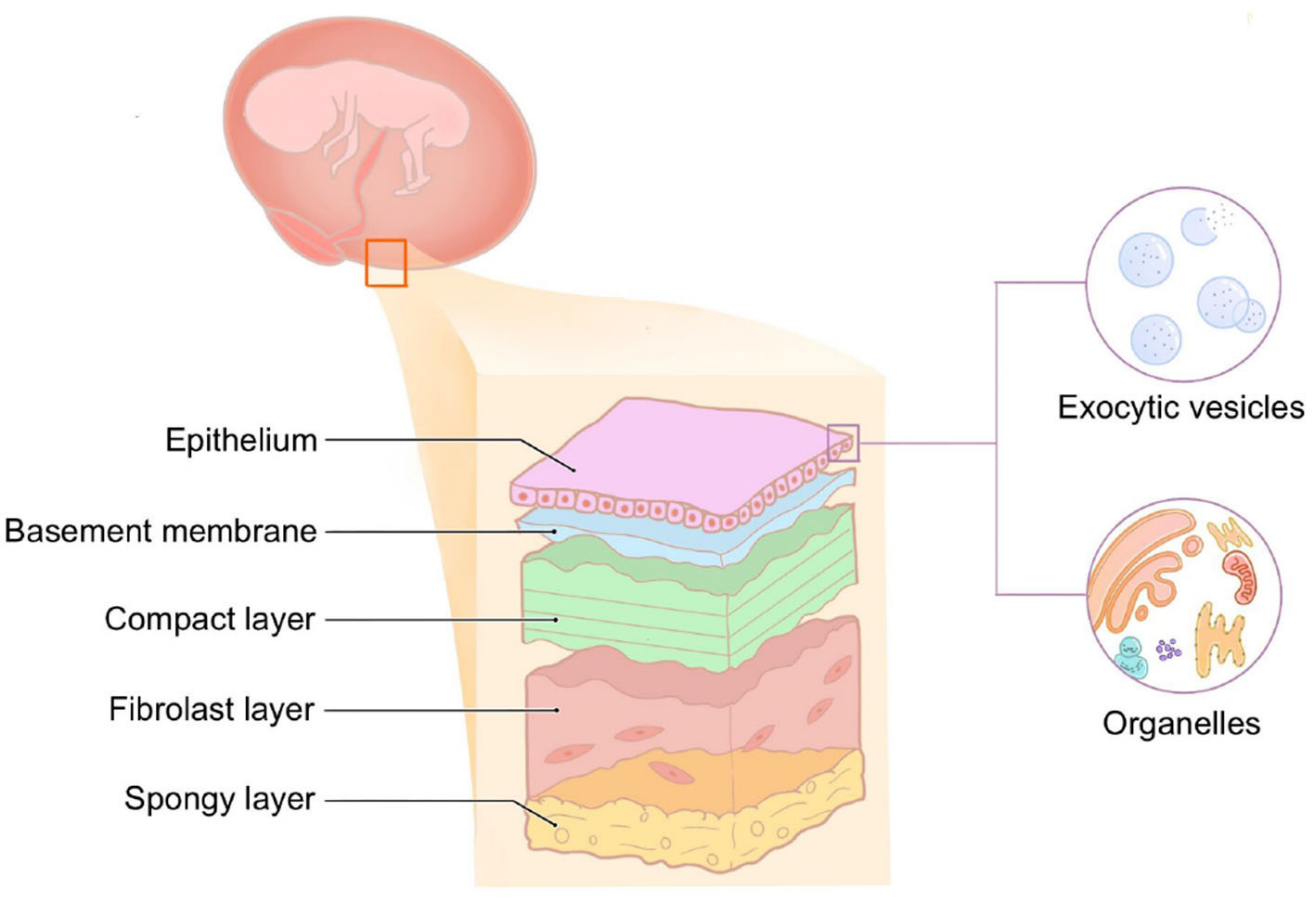
Histological origin and stratification of hAM.

hAM is the innermost layer of the placenta, which is smooth, translucent without blood vessels, nerves, or lymphatic vessels, and has a certain degree of elasticity. Histologically, hAM is a five-layer membrane ranging from 0.02 to 0.5 mm, consisting of the epithelial layer, basement membrane, compact layer, fibroblast layer, and spongy layer (Figure 2). The epithelial layer is of the same origin as the corneal epithelium, which is derived from the ectoderm. It is composed of a monolayer of cuboidal cells abundant in exocytic vesicles and organelles, and there are many microvilli on the surface with active secretory and transport functions. The basement membrane (BM) is the thickest BM in the body, of ~0.1 μm. It contains a large amount of heparan sulfate proteoglycan, which acts as an osmotic barrier for macro-molecules and maintains the integrity of the membrane together with contraction protein, elastin, laminin, etc. The compact layer is cell-free and consists of a dense fibrous network. This layer is rich in type I, type II, and type III collagen and elastin, which imparts AM its tensile strength and elasticity. The tension of AM also depends on the structure of this layer. The fibroblast layer is the thickest and consists of a loose network of fibroblasts within the reticulin matrix. The outermost sponge layer is a transitional layer between the amnion and chorion, consisting of reticulin bundles within a mucin background. Structurally, AM is similar to the conjunctival tissue, a feature that allows for its application in ophthalmology.

## 4. The biological properties of hAM

### 4.1. Anti-inflammation

The anti-inflammatory mechanisms of AM are not yet completely understood. It can act as a physical barrier to inflammatory cell infiltration, attract and trap inflammatory cells, thereby reducing the release of inflammatory mediators. Meanwhile, it can produce secretory leukocyte protease inhibitors (SLPIs) and elastin with anti-inflammatory properties (1). AM also expresses some matrix metalloproteinase (MMP) inhibitors to suppress pathological responses such as inflammation and revascularization where MMPs are involved (2). Additionally, the AM matrix significantly inhibited lipopolysaccharide (LPS)-induced upregulation of IL-1α and IL-1β, which are thought to mediate inflammatory responses. This result may also explain the role of AM transplantation in reducing ocular surface inflammation (3).

### 4.2. Anti-fibrosis

The antifibrotic effect of AM was achieved by inhibiting the transforming growth factor-β (TGF-β) signaling and reducing the expression of TGF-β1, TGF-β2, TGF-β3 isoforms and TGF-β type II receptors. The formation of the scar tissue is mainly due to the excessive proliferation of fibroblasts, and TGF-β can activate fibroblasts. Therefore, the downregulation of the TGF-β signaling system is considered to be a mechanism to prevent scar-forming during the wound healing process. Compared to their counterparts grown in plastic or collagen gel, AM cultures inhibited TGF-β receptor expression in fibroblasts, and this inhibition remained after 1 week of culture, revealing the anti-fibrotic property of AM (4).

### 4.3. Anti-microbial

Studies show that (5), the fetal membrane has an inhibitory effect on various bacteria, including *Hemolytic streptococcus group A, Staphylococcus aureus*, and *Staphylococcus saprophyticus*. Talmi et al. (6) have proven that AM, AM unseparated from the chorion (AMC), and synthetic polyurethane base membrane had an inhibition effect on bacteria cultured in the agar plate. On the one hand, the tight adhesion of the membranes to the agar surface and microorganisms is considered to be the dominant factor for this inhibitory effect. On the other hand, AM expresses several antimicrobial peptides, such as defensin, elastin, and SLPIs, of which β-defensin is the major one expressed by epithelial cells and is essential for the formation of the immune system. These properties validate the eminent antimicrobial properties of AM.

### 4.4. Hypo-immunogenicity

Initially, it was considered that there was no expression of the major histocompatibility antigen, HLA-A, HLA-B, HLA-C, or HLA-DR on the surface of amniotic cells, but further studies have shown that HLA-Ia and HLA-Ib are expressed in trace amounts (7). Kubo et al. (8) further investigated the immunogenicity of cryopreserved AM by transplanting it in the rat corneal stroma, corneal limbus, and renal capsule, respectively. The results showed that no immune response was induced in the cornea (an immune privilege tissue), while only mild cellular infiltration was observed in the corneal limbus and renal capsule (non-immune privilege tissues). AM remained structurally normal with no neovascularization. This experiment demonstrated that, during xenotransplantation, AM did not cause transplant rejection within the immune privilege tissues but only displayed relatively mild immunogenicity in the non-immune privilege tissues.

### 4.5. Anti-angiogenesis

AM can act as a physical barrier to inhibit the proliferation and migration of vascular endothelial cells, thereby preventing neovascularization. Meanwhile, both human amniotic epithelial cells and mesenchymal cells express IL-1 receptor antagonists, four types of TIMPs, type XVIII collagen, and IL-10 (9), all of which are effective anti-angiogenic substances. However, it is to be noted that the anti-angiogenic properties need to be considered in balance with its potential risks (e.g., tissue ischemia) when applied.

### 4.6. Pro-epithelialization

AM may serve as a good extracellular matrix to enhance epithelial adhesion, promote proliferation and migration, and inhibit apoptosis, hence facilitating epithelium formation (10). The extracellular matrix and epithelial layer of AM are scattered with nutrient supplements, particularly epidermal growth factor (EGF), keratinocyte growth factor (KGF), and neurotrophin that supports the aforementioned wound healing properties of the matrix (11). This complex interaction of these components can uniquely regulate and enhance the regenerative healing of the tissue. AM can also take effect on epithelial cell apoptosis through the negative regulation of interleukin-1β-converting enzyme (ICE) expression (12), a feature that manifests its effectiveness in cases of non-healing or persistent epithelial defects.

## 5. The application of hAM in vitreoretinopathy

### 5.1. Retinal hole and detachment

In October 2019, based on the regenerative potential of hAM in repairing the retinal tissue, Rizzo et al. performed hAM transplantation in the subretinal space in six patients with retinal detachment (RD) and retinal hole (RH). One patient had rhegmatogenous retinal detachment (RRD), and five had recurrent RD. All of them had varying degrees of proliferative vitreoretinopathy (PVR), which is considered clinically refractory with a poor prognosis. Three months after the operations, their final BCVA (best corrected visual acuity) improved from 2.33 ± 0.51 logMAR (20/2,000) to 1.2 ± 0.62 logMAR (20/400). Two months after silicone oil (SO) removal, their BCVA improved to 0.8 ± 0.47 logMAR (20/125), with no postoperative adverse events. After a long-term follow-up of more than 1 year, there were no reports of transplant rejection (13).

In November 2019, two cases of plaque chorioretinal atrophy with RD and paravascular RH were treated by Caporossi et al. Both vitrectomy and hAM transplantation into the hole were applied. OCT (optical coherence tomography) imaging showed new tissue growth on hAM graft 2 weeks after the surgery, and it completely covered the hAM plugs 3 months after the surgery. The BCVA increased from 20/2,000 (2 logMAR) to 20/250 (1.1 logMAR). After 2 months of surgery, the SO was extracted, and no recurrence was observed. The patients' corrected vision was stable at 20/250 (14).

In September 2020, Tizio et al. applied an improved surgical procedure using hAM graft and autologous platelet-rich plasma (PRP) transplantation to treat a case of pathological myopia with posterior scleral staphyloma and perivascular RH leading to recurrent RD. hAM graft promoted the healing of RH, and 6 weeks after the operation, an OCT scan showed RH having been covered by new tissues. The SO was removed 3 months later, and no recurrence of RD was observed at postoperative follow-up (15).

In November 2020, six patients with RRD were treated by Saravia et al. using a more easily stored, highly bio-safe, lyophilized AM patch (LAMPatch). Post-operatively, all four patients' LAMPatch fitted well, and the BCVA ranged between 20/30 and 20/100 at 1 week after surgery. LAMPatch tamponade was missing in only one patient after one month, and one patient had partial LAMPatch detachment 5 months after surgery. No recurrence of RD and no complications such as increased IOP, cataract, or inflammation were observed in any patient (16). Detailed information has been summarized in Table 1.

**Table 1:** Results of the amniotic membrane for treatment of retinal holes.

**References**	**Case type**	**Sample size**	**Prognosis BCVA—Snellen (LogMAR)** **(time period)**	**Adverse event** **(number of cases)**
Rizzo et al. (13)	RD with RH	6	20/2,000 (2.33 ± 0.51) to 20/125 (0.8 ± 0.47)	N
			**(5 months)**	
Caporossi et al. (14)	RD with paravascular RH	2	20/2,000 (2.0) to 20/250 (1.1)	N
	Plaque chorioretinal atrophy		**(3 months)**
	Perivascular RH	1		N
Di Tizio et al. (15)	Recurrent RD		20/200 (1.0) to 20/160 (0.9)
	Pathological myopia		**(3 months)**
	Posterior scleral staphyloma		
Saravia et al. (16)	RRD	6	20/30 (0.2)–20/100 (0.7)	LAMPatch not in place (1)
			**(1 week)**	Partially detached (1)

BCVA, best corrected visual acuity; RD, retinal detachment; RRD, rhegmatogenous retinal detachment; RH, retinal hole.

### 5.2. Macular hole

An macular hole (MH) is an anatomic discontinuity of the neurosensory retina that develops in the center of the macula or fovea. Currently, vitrectomy is the most effective treatment for MH. However, in some cases, MH is often difficult to heal due to the large aperture, long courses of the disease, and other complex etiologies. The studies of Fekrat, Funata, and Madreperla (17–19) all indicated that difficult-to-heal MH exhibited varying degrees of active cell proliferation and migration. Therefore, controlling abnormally active cell proliferation and migration become critical to the treatment of refractory MH. Furthermore, AM has anti-inflammatory, anti-fibrotic, and epithelialization-promoting properties. It can provide a favorable homeostasis for cell proliferation and migration, thus enabling the treatment of refractory MH.

In October 2019, Rizzo et al. conducted hAM tamponade and follow-up of eight patients with recurrent MH. After 1 week of the surgery, all patients had an OCT scan showing closure of the holes, with BCVA improved from 1.48 ± 0.49 logMAR (20/800) preoperatively to 0.71 ± 0.37 logMAR (20/800) at 3 months postoperatively and 0.48 ± 0.14 logMAR (20/50) at 6 months postoperatively (13). In April 2021, Saad et al. treated 13 patients with recurrent MH using the same procedure of AM tamponade. In all of the patients, repeated OCT results revealed anatomical closure of the holes, and corrected visions were also improved from 1.7 ± 0.33 (6/300) to 0.9 ± 0.15 (6/48) (20).

Giant MH is also a type of refractory MH. In April 2019, Caporossi et al. performed vitrectomy and AM tamponade in two patients with large MH with complex RD. Both patients had their vision improved from light perception (LP) to 20/400 (logMAR 1.3) at 3 months after surgery. After SO removal at 4 months after surgery, no recurrence of MH was observed, and the visual acuity was stable at 20/400 (21). We have summarized the above information of AM treatment for MH in Table 2.

**Table 2:** Results of the amniotic membrane for treatment of macular holes.

**References**	**Case type**	**Sample size**	**Prognosis BCVA—Snellen (LogMAR)** **(time period)**	**Adverse event** **(number of cases)**
Rizzo et al. (13)	Recurrent MH	8	20/800 (1.48 ± 0.49) to 20/50 (0.48 ± 0.14)	N
			**(6 months)**	
Caporossi et al. (21)	Giant MH	2	20/20,000 (3.0) to 20/400 (1.3)	N
	Complex RD		**(4 months)**	
Saad et al. (20)	Recurrent MH	13	6/300 (1.7 ± 0.33) to 6/48 (0.9 ± 0.15)	N
			**(12 months)**	

BCVA, best corrected visual acuity; RD, retinal detachment; MH, macular hole.

During AM tamponade surgery, because of tissue redundancy, AM larger than the basal diameter of the MH tends to produce folds. In the early phase of the postoperative recovery, inward migration of the retina exacerbates the fold and causes accumulation of the AM tissue at the edge of the hole, preventing further recovery of the hole. Relatively speaking, AM graft that is too small does not come into contact with the edge of the hole and will not induce the closure of it, thus affecting the patient's postoperative vision recovery. In view of this problem, in April 2020, Caporossi et al. (22) proposed that the operator apply OCT to measure the size of the hole prior to the operation. According to the basal diameter of the MH, the diameter of the perforator can be selected and the size of the AM plug be adjusted. Therefore, the recovery of the hole and the improvement of the patient's visual acuity can achieve the best effect postoperatively.

As compared to the inner limiting membrane (ILM) flap insertion for the MH treatment proposed by Chen et al. (23) in 2018, AM tamponade is obviously simpler during the operation. Meanwhile, current clinical studies have not found complications such as rejection or endophthalmitis, and therapeutic RH does not occur compared to ILM flap insertion. In September 2021, Pacini et al. (24) compared the macular micro-structure of recurrent MH after treating the patients with hAM or ILM grafts. Spectral-domain OCT (SD-OCT) imaging of patients receiving hAM implantation showed the recovery of foveal depression, inward growth of tissue above the plugs, and formation of tissue resembling the normal external retina. However, patients with ILM implantation showed a flattened profile, completely different from the normal foveal depression and fiber proliferation. The underlying cellular mechanisms of the fundus tissue remodeling responsible for the difference should be further investigated.

### 5.3. Degenerative diseases

Retinal degenerative diseases (RDD) mainly include age-related macular degeneration (AMD) and retinitis pigmentosa (RP). Current clinical treatment can only delay the progression of such diseases, and there is no clear treatment to reverse retinal degeneration or restore normal retinal function and vision. Retinal cell transplantation holds great potential for treating RDD in future, and retinal pigment epithelium (RPE) transplantation has been shown to rescue photoreceptor cells in dystrophic rats (25). It has also been attempted in patients with dry and wet AMD by two techniques: cell suspension or patch transplantation (26). Cell suspension transplantation may result in random tissue arrangement of multilayer cells in the subretinal space, and various postoperative complications (27). In contrast, patch transplantation not only prevents cell disorganization, excessive mechanical stress at the hole, and cell damage but also ensures the precise positioning of the grafts.

Human retinal pigment epithelium (hRPE) cells transplanted on AM have been shown to adhere to it within 24 h. In addition, the cells maintained their epithelial characteristics (e.g., morphology and pigment) and proliferated. It was also found that hRPE cells growing in the AM were highly organized, which constitute a tight monolayer arrangement with clear cell-cell and cell-substrate interactions (28). These results suggest that the epithelial-free hAM may be the optimal substrate for RPE transplantation into the subretinal space. Similarly, in another stem-cell therapeutic approach considered at the forefront of treating RDD, Nadri et al. (29) found that AM can induce and retain the differentiation potential of mesenchymal stem cells (MSCs) from a trabecular network into photoreceptor cells, making it the ideal substrate in retinal cell transplantation.

AM can also be used as a source of stem cells in stem-cell therapy. Human amniotic epithelial stem cells (hAESC) express several stem-cell markers, such as SSEA-3, SSEA-4, SOX-2, and NANOG, which imparts hAESCs the capability to multi-directional differentiation (30, 31). Pluripotency, low immunogenicity, non-tumorigenicity, easy access, and absence of ethical issues (32, 33) make hAESCs extremely valuable in the clinical treatment of retinal diseases. It has been shown that in preclinical animal models of classical RDD, hAESCs can be induced to photoreceptor-like cells, express their markers, and restore visual function in parts of the subjects (34).

In RDD, AM can also be directly implanted under the retina to play its role. In the study of Rizzo et al. (35) in October 2020, the investigators observed photoreceptor regeneration and recovery after vitrectomy and subretinal AM implantation in 11 patients with damaged RPE. The BCVA increased from 20/2,000 (2 logMAR) to 20/400 (1.31 logMAR). Caporossi et al. (36) then included 26 patients (28 eyes) to further explore the efficacy of subretinal amniotic membrane implantation. The patients were followed up for 9 months, and their best corrected visual acuity improved from an average of 1.9 logMAR before surgery to a final average of 1.2 logMAR. Microperimetry images revealed the presence of photoreceptors on the implanted AM. Caporossi et al. (37) also reported the stable fixation of AM after transplantation.

### 5.4. Optic disc disorder

The optic disc pit is a rare abnormality in the papilla of the optic nerve. Approximately 25–75% of patients will develop a serous detachment of the central macula at some point in their lives, some with an RH, resulting in optic disc pit-associated macular diseases (ODP-M). At present, the pathogenic factors for the disease remain unknown (38).

From July 2018 to January 2019, Rizzo et al. attempted to treat three patients with ODP-M using vitrectomy combined with AM implantation in optic nerve recess and vitreous gas injection. Postoperatively, the patients were found to have decreasing subretinal fluid (SRF), and the BCVA also improved to 20/25 at the 6-month follow-up (39). Subsequently, in February 2022, Caporossi et al. (40) treated 11 patients with ODP-M with subretinal or intraretinal effusion (IRF) using AM implantation in the optic disc recess and extended the follow-up to 12 months. The results showed a significant anatomical improvement in all eyes at 12 months after the treatment. In total, nine of the 11 patients (81.8%) had complete postoperative retinal fluid resorption, and the other two patients (18%) also showed partial retinal fluid resorption. After 12 months, no recurrence of the SRF/IRF fluid was observed, and the mean time to complete resorption was 4.3 ± 1.2 months. The mean visual acuity improved from 20/80 at baseline to 20/32 at the 12-month follow-up. In all cases, the implanted AM exhibited thinning and partial resorption, but it was still detectable in all eyes at the end of the follow-up, and none of the patients experienced any serious complications during the follow-up.

Therefore, alternative biomaterials to seal the optic nerve excavation are the latest popular treatment option, and the ILM has also been applied by researchers to fill the excavation and achieved good results (41). However, compared with the thin and fragile ILM, AM better achieves this effect, without complications associated with the ILM stripping process (e.g., the macular hole). These properties make AM superior to ILM in the treatment of optic disc pits.

Morning glory syndrome (MGS) is also a rare congenital malformation of the optic papilla. It may be a type of optic nerve entrance defect (42); or it may be related to the abnormal development of glia in the center of the optic papilla (43), but the mechanism of this malformation is still unclear. Retinal detachment is a common complication of MGS, which often occurs in one-third of MGS patients (44). However, there are no clear guidelines for the treatment of MGS retinal detachment in clinical practice. Inspired by the recent application of amniotic membrane in vitreoretinal diseases, Caporossi et al. (45) performed vitrectomy with amniotic tamponade in the optic disc in the treatment of patients with macular detachment and retinal detachment. In the first operation, SF6 gas tamponade was performed, but recurrent retinal detachment occurred after gas absorption and the amniotic membrane (3 mm) did not fully cover the defect site. A second vitrectomy with silicone oil filling was therefore performed and a larger amniotic membrane (4 mm) was used. After 3 months of operation, oil extraction revealed a new but smaller retinal detachment, so a third operation was performed and a 6 mm amniotic membrane was used with silicone oil tamponade. After 3 months of surgery, the subretinal fluid completely reabsorbed and the retina fully attached. The best corrected vision reached 20/100.

### 5.5. Choroidal hole

In July 2017, Zhu et al. (46) presented a case of SO migration in the suprachoroidal space associated with a choroidal hole due to trauma. Recurrent RD with PVR occurred in the 4th week after a simple vitrectomy combined with SO tamponade. During the second vitrectomy operation, a choroidal hole of ~3.5 × 4 mm was found in the temporal periphery. An AM plug was used to fill the choroidal hole. After 3 months of surgery, patient's IOP increased from 6 to 10 mmHg after the first surgery. The AM tamponade showed signs of contraction, histogenesis, and neovascularization. The B-scan showed choroidal attachment and a corresponding continuous choroidal echo at the original hole. At the 6-month follow-up examination, the patient was stable with visual acuity improved to 20/300 and an IOP of 12 mmHg. We have summarized the above three sections in Table 3.

**Table 3:** Results of the amniotic membrane for treatment of optic disc disease, regenerative retinal disease, and choroidal hole.

**References**	**Case type**	**Sample size**	**Prognosis BCVA—Snellen (LogMAR)** **[time period]**	**Adverse event** **(number of cases)**
Rizzo et al. (35)	AMD	11	20/2,000 (2.0) to 20 /400 (1.31)	N
			**(6 months)**
Caporossi et al. (36)	AMD	28	20 /2,000 (1.88) to 20/400 (1.23)	N
			**(12 months)**	
Rizzo et al. (39)	ODP-M	3	20/40 (0.333) to 20/25 (0.097)	N
			**(6 months)**	
Caporossi et al. (40)	ODP-M	11	20/80 (0.58 ± 0.2) to 20/32 (0.16 ± 0.08)	N
			**(12 months)**
Caporossi et al. (45)	MGS	1	20/100 (0.699) to 20/100 (0.699)	N
			**(6 months)**
Zhu et al. (46)	Choroidal hole	1	NLP (3.505) to 20/300 (1.176)	N
			**(6 months)**	

ODP-M, Optic disc pit-associated macular diseases; MGS, morning glory syndrome; AMD, age-related macular degeneration; NLP, no light perception.

## 6. Discussion

In recent years, more and more researchers are expanding the application of hAM to the posterior segment of the eye, making it a popular field of research. Due to the excellent biological properties of hAM, its application in the posterior segment has shown satisfactory results. However, owing to the limited sample size, most of the current studies are confined to clinical outcomes, and the mechanism is not yet clarified. There are no comparative reports with other emerging surgical studies. Therefore, more prospective, multicenter randomized controlled studies should be carried out to confirm these aspects.

Although the application effect of hAM in the direction of RH and MH is promising, the current study alone cannot explain whether the holes are sealed by the regeneration of the retinal neuroepithelial layer. Moreover, in some reports, the operation has been associated with the disappearance and displacement of hAM, so how to avoid these occurrences is a major concern in future. It is also necessary to further explore the mechanism of cell growth and the growth orientation after AM tamponade as well as which side of the AM adhesion is more beneficial to the recovery of the macular and retinal structure during the operation.

The current study has explored three aspects of hAM as a substrate, implant, and stem cell source. Although hAM can develop toward photoreceptor cells *in vitro* as a substrate or as a source of stem cells, when implanted directly under the retina, the mechanism is not clear. Whether hAM functions as a substrate promoting the development of photoreceptor cells or hAM's stem cells develop toward photoreceptor cells deserves further investigation. The advantages and disadvantages of the above three treatment options are also worthy of comparison in clinical studies.

The hAM implantation in the treatment of optic disc pits is thought to have structurally closed the pits, thereby reducing SRF/IRF fluid, but the specific mechanism is still unclear. There is also a lack of research on whether hAM can remain in this closed structure intraocularly for long periods.

At present, there is only one case of hAM transplantation for the choroidal hole, which is still in the exploratory stage. More research is needed to probe the repairing ability and to study the repairing mechanism of hAM on the choroidal hole in comparison with other therapeutic methods, such as scleral buckling.

## 7. Conclusion

In conclusion, the application of hAM in the posterior segment of the eye has gradually become a heated field of research. Its superb biological properties and structures present great potential in fields such as retinal hole, macular hole, optic diseases, retinal regenerative disease, and choroidal hole. It deserves further exploration in more prospective and larger randomized controlled studies to evaluate its underlying applications in the posterior segment of the eye.

## Author contributions

HY, ZL, and WJ: study conceptualization, methodology, and original draft preparation. ZL: review and editing and figures preparation. WJ and AY: supervision. All authors have read and agreed to publish the manuscript.
